# A Systematic Investigation of Computation Models for Predicting Adverse Drug Reactions (ADRs)

**DOI:** 10.1371/journal.pone.0105889

**Published:** 2014-09-02

**Authors:** Qifan Kuang, MinQi Wang, Rong Li, YongCheng Dong, Yizhou Li, Menglong Li

**Affiliations:** 1 College of Chemistry, Sichuan University, Chengdu, China; 2 College of Computer Science, Sichuan University, Chengdu, China; 3 College of Life Science, Sichuan University, Chengdu, China; Huazhong University of Science and Technology, China

## Abstract

**Background:**

Early and accurate identification of adverse drug reactions (ADRs) is critically important for drug development and clinical safety. Computer-aided prediction of ADRs has attracted increasing attention in recent years, and many computational models have been proposed. However, because of the lack of systematic analysis and comparison of the different computational models, there remain limitations in designing more effective algorithms and selecting more useful features. There is therefore an urgent need to review and analyze previous computation models to obtain general conclusions that can provide useful guidance to construct more effective computational models to predict ADRs.

**Principal Findings:**

In the current study, the main work is to compare and analyze the performance of existing computational methods to predict ADRs, by implementing and evaluating additional algorithms that have been earlier used for predicting drug targets. Our results indicated that topological and intrinsic features were complementary to an extent and the Jaccard coefficient had an important and general effect on the prediction of drug-ADR associations. By comparing the structure of each algorithm, final formulas of these algorithms were all converted to linear model in form, based on this finding we propose a new algorithm called the general weighted profile method and it yielded the best overall performance among the algorithms investigated in this paper.

**Conclusion:**

Several meaningful conclusions and useful findings regarding the prediction of ADRs are provided for selecting optimal features and algorithms.

## Introduction

Early and accurate identification of ADRs is critically important for drug development and clinical safety. Traditional clinical trials to recognize ADRs are expensive and time-consuming. Conversely, computer-aided methods for predicting ADRs are much cheaper and quicker than clinical trials and highly reliable [1]–[3].

**Figure 1:**
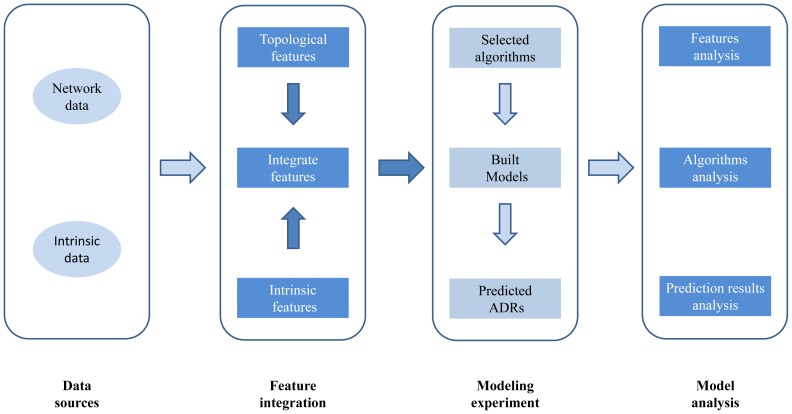
Overview of the main workflow in this paper. First, data were integrated from multiple sources, including network data (drug-ADR associations) and intrinsic data (chemical structures and ATC taxonomies of drugs and MedDRA taxonomies of ADRs). Next, topological features and intrinsic features were constructed based on network data and intrinsic data, respectively, and then integrated features were constructed by integrating topological features with intrinsic features, Finally different algorithms were selected to construct models to predict ADR, and comparative analyses were performed for features, algorithms and prediction results based on modeling experiments.

Constructing machine learning models by combining intrinsic features of drugs and ADRs with topological features of drug-ADR association networks has been one of typical computer-aided methods for predicting ADRs [4], [5]. However, many other state-of-the-art methods have been proposed to predict drug targets [6]–[15]. Computer-aided prediction of drug targets is similar to prediction of ADRs: there are close relationships between ADRs and drug targets that have been identified in biological systems [16], [17]. In addition, in terms of mathematics, the prediction of ADRs and drug targets can both be abstracted into link prediction models on a bipartite network; therefore most of the computational processing steps are similar between these two systems. We therefore hypothesize these series of state-of-the-art methods, which have been successfully applied in the prediction of drug targets, could also achieve excellent performance in the prediction of ADRs. Our results also support this hypothesis indirectly. Hence, in recent years, many computational methods have been proposed to predict ADRs or drug targets, whereas less attention has been paid to compare and analyze existing computational methods and features. Here, we summarize the existing computation methods and features that have been proposed, extract classical methods and features to construct different representative computational models for predicting ADRs, and compare and analyze these methods and features. Finally, useful findings are provided for searching optimal features, appropriate algorithms for predicting ADRs. A brief illustration of the main workflow in this paper is shown in **Figure 1**.

## Materials and Methods

### Materials

In this paper, two drug-ADR association networks were constructed; one was called the training network, and the other was called the testing network.

To construct the training network, drug data were collected from the following databases: DrugBank [18], Kegg [19], FDA Adverse Event Reporting System (FAERS, website: www.fda.gov/Drugs/GuidanceComplianceRegulatoryInformation/Surveillance/AdverseDrugEffects/default.htm) of 2005, and SIDER [20]. To reduce the proportion of false positives in drug-ADR associations from SIDER and FAERS (in 2005), an interacting drug-ADR pair was taken only when the drug-ADR pair was recorded in both databases. In addition, according to the Medical Dictionary for Regulatory Activities (MedDRA) [21], ADRs can be divided into five different levels: the System outraged Class (SOC), the High Level Group Term (HLGT), the High Level Term (HLT), the Preferred Term (PT), and the Lowest Level Term (LLT). Here, only ADRs in the HLT Level were considered; therefore, ADRs recorded in FAERS and SIDER that belonged to PT or LLT were first mapped to HLT.

**Figure 2:**
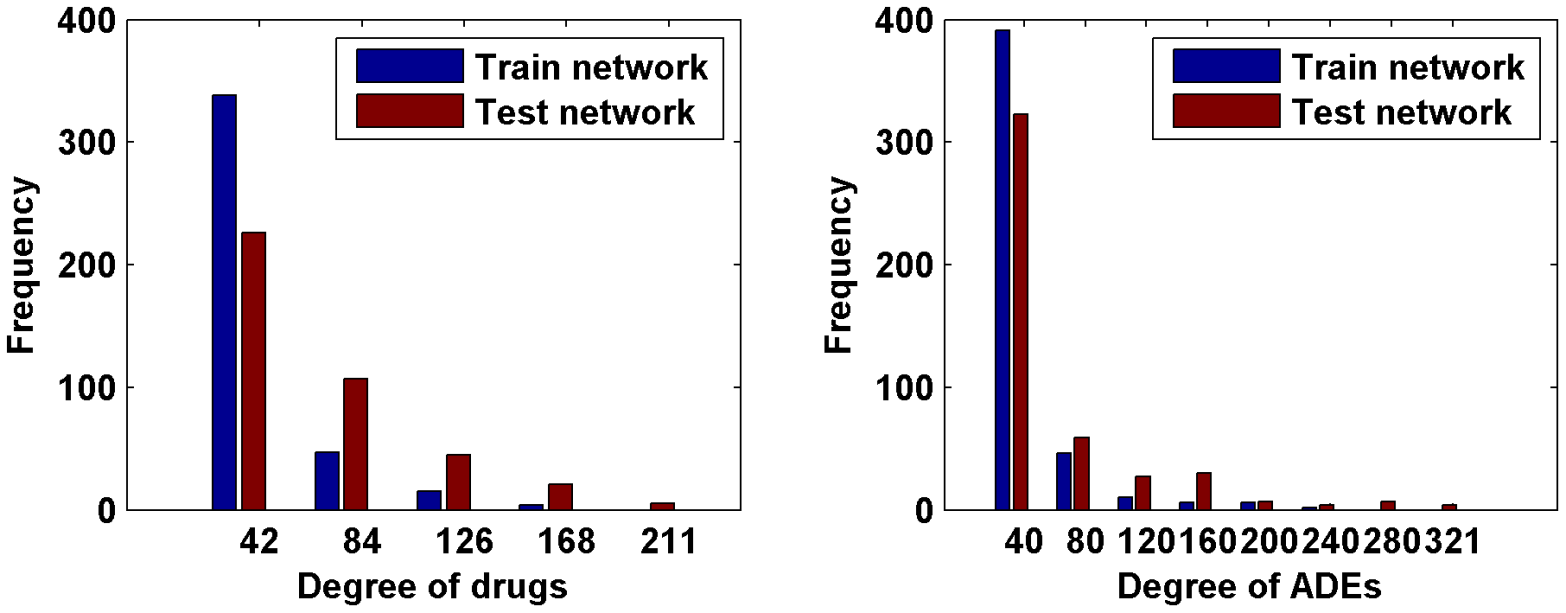
Degree distributions of drugs and ADRs. The left panel depicts the histograms of the degrees of drugs. The right panel depicts the histograms of the degrees of ADRs.

We obtained the testing network by adding drug-ADR associations recorded in both FAERS and SIDER from 2006 to 2011 to the training network. Finally, the network node sets (consisting of drug nodes and ADR nodes) were identical in the training and testing networks, whereas the network edge sets (interacting drug-ADR pairs) were different. The related quantitative statistics of the drug-ADR networks are provided in **Table 1** and **Figure 2**.

**Table 1:** Statistics for the drug-ADE networks.

Statistics	Train drug-ADE network	Test drug-ADE network
Number of drugs	404	404
Number of ADEs	461	461
Number of drug-ADE associations	9180	19182
Average degree of drugs	22.7	47.5
Average degree of ADEs	19.9	41.6

### Problem formalization

The problem of predicting ADRs of drugs can be abstracted to the problem of predicting new interactions in a drug-ADR association network. Formally, 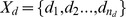 and 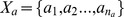 represent a set of the drug nodes and ADRs nodes in a drug-ADR association network, respectively, and the edges in the network represent interacting drug-ADR pairs. Furthermore, this bipartite network can be characterized as an 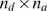 adjacency matrix Y. That is, 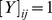 if an existing association is previously known between 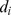 and 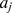, and 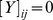 otherwise. In addition, to make it more convenient for later description, the set of prediction scores for each drug-ADR pair are characterized as an 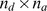 matrix 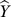, where the element 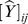 represents the prediction score of the drug-ADR pair 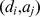. The set of similarity scores of drugs and similarity scores of ADRs are characterized as an 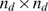 similarity matrix 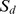 and an 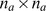 similarity matrix 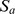, respectively. The elements 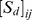 and 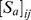 represent the similarities of the drug-drug pair 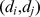 and the ADR-ADR pair 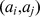, respectively. One of main tasks in this paper was to compute the prediction score of each non-interacting drug-ADR pair 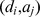 and then to determine whether an association between 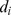 and 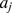 existed using the prediction score of the drug-ADR pair 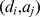.

### Model features

Features of drugs or ADRs in this paper were used to characterize the similarity of drugs or ADRs. Here, intrinsic features and topological features of drugs' and ADRs were employed.

#### Topological feature

To extensively investigate the effect of topological features on computational models for predicting ADRs, six common topological features and a new topological feature designed by us were employed to characterize the similarity of drugs or ADRs.

1. Jaccard coefficient (denoted 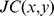): 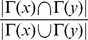. Here, 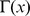 and 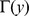 represent the neighborhood set of homology nodes x and y, respectively. In drug-ADR association network, there are two classes of nodes (drug nodes or ADR nodes). Therefore, the relationship of any two drug (ADR) nodes is homologous, while, the relationship between a drug node and an ADR node is heterologous, here, if two nodes both belong to drug or ADR nodes, we call them as homology nodes. In addition, the symbol 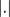 represents the number of elements in a set.
2. Gaussian interaction profile kernel (denoted 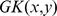): this feature is proposed in by the scholar Laarhoven and has been successfully applied to predict drug-target interactions [12].
3. A topological feature proposed by the scholar Allali [22] (denoted 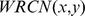): 
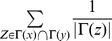
;
4. Neighbors Product (denoted 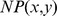): 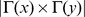;
5. Common Neighbors (denoted 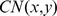): 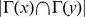;
6. A feature proposed by the scholar L.A.Adamic [23] (denoted 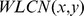): 
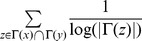
;
7. A new feature is designed by us, which is similar to 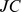 (denoted 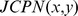): 
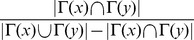
;

#### Intrinsic features

The intrinsic features were obtained from chemical structures or biological functions of drugs or ADRs. The intrinsic features of drugs were based on chemical structures and the ATC taxonomy of drugs [24], [25], and the intrinsic features of ADRs were based on the MedDRA taxonomy of ADRs. The chemical similarities between drugs were computed using SIMCOMP [26], and the ATC taxonomy similarities between drugs and the MedDRA taxonomy similarities between ADRs were both computed using the semantic similarity algorithm [5], [11], [27].

### Classification algorithm

There are many state-of-the-art methods to predict drug targets. In this study, we selected the regularized least – squares classifier, semi-supervised link prediction classifier and the nearest – neighbor classifier from these existing methods to predict ADRs. There are several justifications for this selection. The performance of methods [7], [8], [9], [12], [13], [14] have been tested on a same dataset [7], the performance of method [12] based on the regularized least – squares and method [9] based on the semi-supervised link prediction was competitive with others, especially, method [12] yielded the highest performance among these methods. On the other hand, regularized least – squares classifier, semi-supervised link prediction classifier and the nearest – neighbor classifier belong to supervised learning, semi-supervised learning and memory-based algorithm, respectively, therefore, these three classifiers were representative of different classes of algorithms among existing methods. We briefly discuss these algorithms below.

#### RLS

The Regularized Least-Squares classifier (denoted RLS) [12], [28] is a basic supervised learning algorithm. If an appropriate kernel has been chosen for RLS, the accuracy of RLS will be similar to support vector machine (SVM), whereas the computation complexity of RLS is much less than SVM. The RLS algorithm can be divided into three separate sub algorithms for defining the kernel matrix: RLS-KP, RLS-KS and RLS-avg. Here, KP and KS are short for Kronecker Product [25], [29] and Kronecker Sum [29], respectively.

#### SLP

Semi-supervised Link Prediction classifier (denoted SLP) is a semi-supervised learning algorithm [9], [30], and the basic assumption of SLP is “Two node pairs that are similar to each other are likely to have the same link strength” [30]. Based on this assumption, the objective function is defined as:
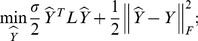
where 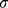 is a regularization parameter and 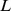 is a Laplacian matrix. SLP also can be divided into three independent sub algorithms for defining 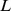: SLP-KP, SLP-KS and SLP-avg.

#### NN

The Nearest-Neighbor classifier (denoted NN) is a simple memory-based algorithm (more detailed descriptions regarding algorithms are provided in **File S1**).

## Results and Discussion

### Evaluation

Ten-fold cross validation and prospective evaluation were used to evaluate the performance of each model. For ten-fold cross validation, interacting drug-ADR pairs and non-interacting drug-ADR pairs were each randomly divided into ten folds of roughly equal size; in each run of the method, one fold of interacting drug-ADR pairs and one fold of non-interacting drug-ADR pairs were left out by setting their entries in adjacency matrix Y to 0. We then attempted to recover their true labels using the remaining data. Note that the Y matrix corresponds to the training network. For prospective evaluation, the training data consisted of the training network and the validation data consisted of all the testing network drug-ADR pairs that were non-edges in the training network. We attempted to recover the true labels of the validation using the training network.

We assessed the model performance with the following two common quantitative indexes: AUC [31] and AUPR [32]. The value of AUC is determined from the area below a curve relating the proportion of true positives versus the proportion of false positives, whereas the value of AUPR is determined from the area below a curve relating precision versus recall.

### Feature analysis

In this paper, two types of features (topological features and intrinsic features) were employed in the modeling experiment. To comprehensively analyze these features, associations between features were first investigated, and then the performances of models constructed using only intrinsic features or topological features were tested, and lastly, the performances of models constructed with integrated features were evaluated.

### Associations between features

Here, Pearson correlation coefficients among drug or ADR features were calculated separately. The detailed results are listed in **Table S1** and **Table S2**. The Pearson coefficients among drug and ADR features were consistent. The intrinsic features of drugs or ADRs were not significantly correlated with topological features of drugs or ADRs, indicating that the information of topological features and intrinsic features may be complementarily used in a prediction model.

### Modeling with intrinsic features

Within the intrinsic features, the chemical similarity of drugs, ATC similarity of drugs and MedDRA similarity of ADRs were denoted by 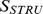, 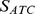 and 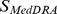, respectively. The Pearson coefficient between 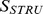 and 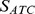 was 0.0905, indicating no significant association between 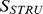 and 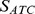. Here, 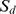 is defined by integrating 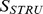 with 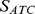 as follows: 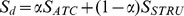, where 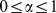; and 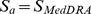. In the modeling experiments, ten-fold cross validation and the Grid Search Method [33] were used to obtain the optimal value of 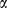. The detailed results are listed in **Table S3** and **Table S4**: when 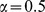, the model achieved slight better overall performance than other models.

### Modeling with topological features

The process of modeling with topological features was similar to as with intrinsic features. Here, seven topological features were respectively used to construct models. The detailed results are listed in **Table 2** and **Table 3**. Almost all models built with the topological feature 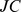 yielded good performance (except SLP-KP). Hence, compared with the other six topological features, 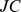 has the most important and general effect on predicting drug-ADR associations.

**Table 2:** AUC scores of the models built with different topological features.

	AUC						
	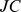	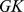	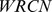	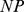	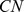	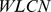	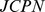
RLS-KP	91.1(0.1)	92.7(0.1)	80.9(0.1)	90.2(0.1)	50.8(0.3)	48.8(0.5)	50.5(0.7)
RLS-KS	92.1(0.1)	93.3(0.1)	89(0.1)	91.2(0.1)	68.4(0.2)	54.4(0.2)	50.4(1.0)
RLS-avg	93.1(0.2)	92.7(0.1)	91.9(0.2)	91.1(<0.1)	72.6(0.2)	63.5(0.4)	52.2(3.0)
SLP-KP	43.2(2.4)	48.6(1.4)	48.3(5.6)	9.8(0.1)	49.5(1.1)	49.7(0.8)	49.5(0.4)
SLP-KS	91.1(<0.1)	34.4(0.1)	30.9(4.5)	8.7(0.1)	49(3.7)	49.7(2.1)	85.9(3.0)
SLP-avg	93.1(<0.1)	90.7(<0.1)	93.2(<0.1)	91.9(<0.1)	92.7(<0.1)	92.6(<0.1)	93.1(0.1)
NN	92.1(0.1)	36.2(0.1)	90.9(0.1)	89.4(0.1)	89.9(<0.1)	89.7(<0.1)	91.3(0.8)
GWPM	93.0(0.1)	90.7(<0.1)	93.0(<0.1)	91.0(0.1)	92.9(0.1)	92.8(0.1)	93.6(<0.1)

Determined from ten-fold cross validation experiments. The AUC scores are normalized to 100.

**Table 3:** AUPR scores of the models built with different topological features.

	AUPR						
	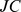	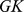	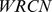	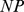	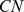	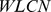	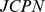
RLS-KP	63.5(0.2)	60.4(0.2)	38.5(0.3)	53.1(0.1)	5.9(0.1)	4.7(<0.1)	5.3(0.3)
RLS-KS	63.6(0.1)	58.7(0.1)	45.6(0.2)	52.8(0.1)	10.5(0.1)	6(0.1)	5.8(0.2)
RLS-avg	63.4(0.1)	58.4(0.1)	59(0.2)	53.2(<0.1)	14.2(0.1)	7.3(0.1)	7.2(0.9)
SLP-KP	4.9(0.5)	4.1(0.5)	20.5(5.3)	3.6(2.2)	6.3(0.2)	5.5(0.2)	4.9(0.4)
SLP-KS	51.7(0.1)	3.9(0.7)	10(0.4)	2.6(<0.1)	10.1(1.5)	7.3(0.6)	26.9(5.4)
SLP-avg	57.7(0.1)	30.1(<0.1)	57.4(0.1)	53.5(<0.1)	56.1(0.1)	55.9(0.1)	58.1(0.1)
NN	52.1(0.2)	4.1(<0.1)	41.7(0.1)	36.4(0.1)	39.5(0.1)	39.1(0.2)	51.9(0.2)
GWPM	60.0(0.1)	30.9(<0.1)	59.5(0.1)	53.2(0.1)	58.1(0.1)	57.4(0.1)	65.4(0.2)

Determined from ten-fold cross validation experiments. The AUPR scores are normalized to 100.

### Modeling with integrated features

Here, the features that integrate topological features with intrinsic features were further investigated. The intrinsic similarity matrices of drugs and ADRs were defined as 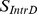 and 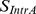, respectively (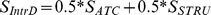; 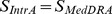). The integrated features were as follows: 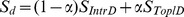; 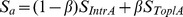; where 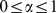, 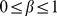, and the topological features of drugs and ADRs were denoted as 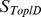 and 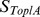, respectively. In the modeling experiments, ten-fold cross validation and the Grid Search Method were used to obtain the optimal values of 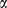 and 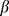 for each integrated feature. The detailed results are delineated in **Table S5**, **Table S6**, **Table S7** and **Table S8**. Compared with models constructed with intrinsic or topological features separately, models constructed with integrated features yielded better performance; that is, the information of intrinsic features and topological features was complementary.

### Algorithm analysis

According to the above results, the best performance of models was obtained from RLS-avg with an optimal integrated feature that integrated 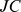 with intrinsic features of drugs and ADRs ([AUC,AUPR] = [0.933,0.635]). While, models constructed using SLP-avg with either intrinsic features or topological features of drugs and ADRs all yielded excellent performance. Therefore, among these seven sub algorithms, models constructed using SLP-avg yielded the best overall performance, demonstrating that SLP-avg is a more general algorithm for predicting drug-ADR associations.

By comparative analysis of the structure of each algorithms, final formulas of these algorithms could be unified as: 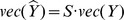 or 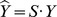; here, 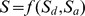, where 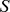 is a function of the similarity matrices 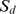 and 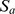, and 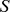 is a symmetric matrix. More detailed descriptions of the unified formulas are provided in the **File S2**. For unify formulas, 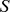 was considered as a similarity matrix of drug-ADR pairs, therefore, all models in this paper can be converted to simple linear models, and the major difference between these models occurs in methods regarding the construction of 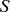. Based on the above analysis, we attempted a simple general linear method to construct 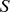 and then designed a simple algorithm called general weighted profile method (denoted GWPM, a more detail description of GWPM is provided in **File S1**) And the performance of this algorithm of prediction ADRs by ten-fold cross validation was shown in **Table 2**, **Table 3**, **Tables S5**, **Tables S6**, **Tables S7** and **Tables S8**. Although the computation complexity of GWPM is relatively lower than other algorithms (except NN), the overall performances of models constructed using GWPM was even better than SLP-avg, especially, the model constructed using GWPM with the optimal integrated feature integrating 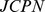 with intrinsic features yielded the best performance ([AUC, AUPR] = [0.942, 0.657]) among all test models in this paper. Hence, finding a good method for constructing 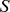 (which is equivalent to finding a proper mapping function from drug and ADR space to drug-ADR pair space) is the key to predicting of drug-ADR associations.

### Statistical analysis of model predictions

**Figure 3:**
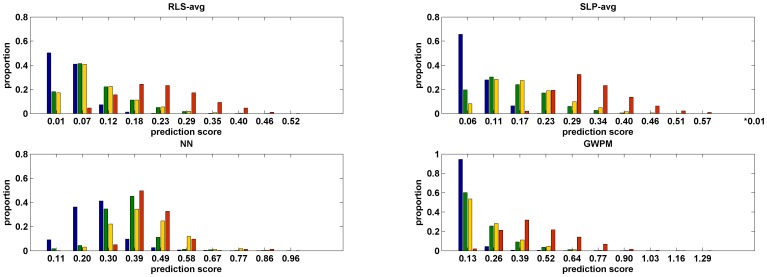
Distribution of prediction scores for different types of drug-ADR pairs. The histograms of distributions of prediction scores of models built by four algorithms are shown. In each sub panel, the blue, green, yellow and red histograms represent the distributions of prediction scores for low degree drug- low degree ADRs, high degree drug- low degree ADRs, low degree drug- high degree ADRs and high degree drug- high degree ADRs, respectively.

According to the above results regarding model performance based on ten-fold cross validation, models were rebuilt by each algorithm with the optimal feature and then validated by prospective evaluation. For RLS and SLP, we selected one sub algorithm among the three sub algorithms (RLS-avg and SLP-avg, respectively), and the detailed results are presented in **Table 4**. The associations between prediction scores of drug-ADR pairs and degrees of drugs or ADRs were also investigated. If the degree of drug or ADR was more than 40 in the training network, then the drug or ADR was considered as a high degree drug or ADR; otherwise, was considered as a low degree drug or ADR. Hence, all drug-ADR pairs were divided into four types: low degree drug- low degree ADR pair, high degree drug- low degree ADR pair, low degree drug- high degree ADR and high degree drug- high degree ADR. The prediction score distribution of these four type drug-ADR pairs is shown in **Figure 3**. Drug-ADR pairs that had known interactions in the training network were not recorded in the prediction score distribution. According to **Figure 3**, the prediction scores of drug-ADR pairs and degrees of drugs or ADRs displayed positive correlations, indicating that the interaction between drug-ADR pairs containing high degree drugs or ADRs were more likely to be predicted correctly by models. Each model has limited ability to predict low degree drug- low degree ADR associations. On one hand, this result demonstrated the limitation of topological features; on the other hand, although integrated features have integrated topological and intrinsic features, the limitation of topological features was not compensated sufficiently well by intrinsic features. Therefore, more effective intrinsic features of drugs and ADRs still require further investigation to improve the model prediction performance.

**Table 4:** The performances of the optimal models validated by prospective evaluation.

		Weight coefficient		Model performance	
Algorithm	feature	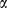	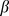	AUC	AUPR
RLS-avg	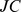	0.9	1	79.2	25.5
SLP-avg	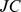	0.9	0.7	85.1	26.4
NN	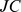	0.7	1	83.1	26.1
GWPM	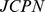	0.8	0.8	82.7	26.9

Here, 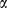 indicates the weight coefficient of drug topological features, 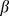 indicates the weight coefficient of ADR topological features; The AUC and AUPR scores are normalized to 100.

#### Comparative with other existing ADR prediction literature

We are aware of only a few other studies that attempts to predict unknown likely ADRs through combining intrinsic and topological features methods [4], [5]. The study [5] and the current study are similar in that they both integrate various types of information to predict unknown likely ADRs, and conclusions about various features are consistent. The data and methods used by the two studies differ in several ways. In current study, drug-ADR associations were extracted from following databases: FAERS and SIDER, and to reduce false positives in drug-ADR associations, a drug-ADR pair was taken only when it was recorded in both databases. While, in study [5], drug-ADR associations were mainly extracted from a proprietary commercial database widely used in hospitals today, provided by Lexicomp (http://www.lexi.com). Perhaps the most important distinction between these two studies lies in computational methods for predicting ADRs. Seven different methods were used in current paper (six methods had been used for predicting drug targets before, and one methods proposed by ourselves) and a systematic comparative analysis is conducted in terms of performance of these methods, finally, some general conclusion regarding algorithms and features is obtained, such as, the feature Jaccard coefficient had an important and general effect on the prediction of drug-ADR associations, final formulas of algorithms selected in current study were all converted to linear model in form. Compared with [5], which only used a logistic regression predictive model. In order to facilitate benchmark comparisons between methods in two studies, we tested the performance of the method used in study [5] on data sets used in current paper, and performance evaluated by ten-fold cross validation and prospective evaluation are [AUC, AUPR] = [0.927, 0.616] and [AUC, AUPR] = [0.793, 0.249], respectively. While, in the current paper, for example, the best performance of GWPM evaluated by ten-fold cross validation and prospective evaluation are [AUC, AUPR] = [0.942, 0.657] and [AUC, AUPR] = [0.827, 0.269], respectively. The results showed performance of methods used in current study was competitive with the study [5].

#### Conclusions

In this paper, three typical algorithms and a new algorithm combining ten features were used to construct models to predict new drug-ADR associations. Different algorithms, features and prediction results were compared and analyzed respectively. Finally, several meaningful conclusions were drawn as follows:

Seven topological features and three intrinsic features of drugs or ADRs were analyzed in this paper. Among these seven topological features 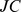 had the most important and general effect on the prediction of drug-ADR associations. In addition, models built using integrated features had better performance than using only topological or intrinsic features, demonstrating that topological and intrinsic features were complementary. However, for rare ADRs (only a few drugs have been currently validated to have these ADRs), models built with integrated features did not correctly predict associations between these ADRs and drugs. Therefore, more effective intrinsic features of drugs and ADRs still require further investigation.

GWPM yielded the best overall performance among all algorithms in this paper as determined from ten-fold cross validation. Additionally, because all algorithms have unified linear formulas, finding an optimal method for constructing the similarity coefficient matrix in the linear formula will be useful to improve accuracy of predicting drug-ADR associations.

## Supporting Information

Table S1 **Associations between drug feature covariates.**
(DOC)

Table S2 **Associations between ADR feature covariates.**
(DOC)

Table S3 **AUC scores of models built with different intrinsic features.**
(DOC)

Table S4 **AUPR scores of models built with different intrinsic features.**
(DOC)

Table S5 **AUC scores of models built with optimal integrated features.**
(DOC)

Table S6 **AUPR scores of models built with optimal integrated features.**
(DOC)

Table S7 **Drug features weight coefficients of models built with optimal integrated features.**
(DOC)

Table S8 **ADR features coefficients of models built with optimal integrated features.**
(DOC)

File S1 **Supplementary Algorithms.** More detailed descriptions regarding algorithms have been provided in this file.
(DOC)

File S2 **Supplementary Algorithm Analysis.** More detailed descriptions of the unified formulas of algorithms have been provided in this file.
(DOC)

File S3 **MATLAB source code.** This file includes MATLAB resource code to implement computational experiments in this paper.
(PDF)

File S4 **This file lists all drug-ADR association pairs in the training network.**
(XLSX)

File S5 **This file lists all drug-ADR association pairs in the testing network.**
(XLSX)

File S6 **This file lists the chemical structure similarity score of each drug-drug pair.**
(TXT)

File S7 **This file lists the ATC similarity score of each drug-drug pair.**
(TXT)

File S8 **This file lists the MedDRA similarity score of each ADR-ADR pair.**
(TXT)
