# Supplementary material for: A Systematic Investigation of Computation Models for Predicting Adverse Drug Reactions (ADRs)
Source: PLoS One. 2014 Sep 2;9(9):e105889. doi: 10.1371/journal.pone.0105889 (PMC4152017; doi:10.1371/journal.pone.0105889)
Supplement: Table S1 — Associations between drug feature covariates. (DOC) [file pone.0105889.s001.doc]

**Table S1.Associations** between drug feature covariates

| Drug feature | $S_{JC}$ | $S_{GK}$ | $S_{WRCN}$ | $S_{NP}$ | $S_{CN}$ | $S_{WLCN}$ | $S_{JCPN}$ | $S_{STRU}$ | $S_{ATC}$ |
| --- | --- | --- | --- | --- | --- | --- | --- | --- | --- |
| $S_{JC}$ | 1 |  |  |  |  |  |  |  |  |
| $S_{GK}$ | -0.1365 | 1 |  |  |  |  |  |  |  |
| $S_{WRCN}$ | 0.5161 | -0.2300 | 1 |  |  |  |  |  |  |
| $S_{NP}$ | 0.4775 | -0.4297 | 0.7621 | 1 |  |  |  |  |  |
| $S_{CN}$ | 0.6846 | -0.4440 | 0.8000 | 0.9331 | 1 |  |  |  |  |
| $S_{WLCN}$ | 0.7001 | -0.4646 | 0.7568 | 0.9182 | 0.9964 | 1 |  |  |  |
| $S_{JCPN}$ | 0.8441 | -0.1759 | 0.4148 | 0.5123 | 0.6813 | 0.7033 | 1 |  |  |
| $S_{STRU}$ | 0.1780 | 0.0436 | 0.1283 | 0.0324 | 0.0685 | 0.0612 | 0.0273 | 1 |  |
| $S_{ATC}$ | 0.1458 | 0.0266 | 0.1090 | 0.0244 | 0.0516 | 0.0452 | -0.0108 | 0.0905 | 1 |
