# Supplementary material for: A Systematic Investigation of Computation Models for Predicting Adverse Drug Reactions (ADRs)
Source: PLoS One. 2014 Sep 2;9(9):e105889. doi: 10.1371/journal.pone.0105889 (PMC4152017; doi:10.1371/journal.pone.0105889)
Supplement: Table S2 — Associations between ADR feature covariates. (DOC) [file pone.0105889.s002.doc]

**Table S2.Associations** between ADR feature covariates

| ADR feature | $S_{JC}$ | $S_{GK}$ | $S_{WRCN}$ | $S_{NP}$ | $S_{CN}$ | $S_{WLCN}$ | $S_{JCPN}$ | $S_{MedDRA}$ |
| --- | --- | --- | --- | --- | --- | --- | --- | --- |
| $S_{JC}$ | 1 |  |  |  |  |  |  |  |
| $S_{GK}$ | -0.1509 | 1 |  |  |  |  |  |  |
| $S_{WRCN}$ | 0.4886 | -0.2050 | 1 |  |  |  |  |  |
| $S_{NP}$ | 0.4443 | -0.2924 | 0.8872 | 1 |  |  |  |  |
| $S_{CN}$ | 0.5918 | -0.3346 | 0.9048 | 0.9521 | 1 |  |  |  |
| $S_{WLCN}$ | 0.6046 | -0.3556 | 0.8709 | 0.9393 | 0.9969 | 1 |  |  |
| $S_{JCPN}$ | 0.5565 | -0.1336 | 0.3772 | 0.3752 | 0.4636 | 0.4718 | 1 |  |
| $S_{MedDRA}$ | 0.2926 | 0.0446 | 0.1053 | 0.0196 | 0.0647 | 0.0586 | 0.0009 | 1 |
