# Supplementary material for: A Systematic Investigation of Computation Models for Predicting Adverse Drug Reactions (ADRs)
Source: PLoS One. 2014 Sep 2;9(9):e105889. doi: 10.1371/journal.pone.0105889 (PMC4152017; doi:10.1371/journal.pone.0105889)
Supplement: Table S3 — AUC scores of models built with different intrinsic features. (DOC) [file pone.0105889.s003.doc]

**Table S3**.AUC scores of models built with different intrinsic features

|  | AUC | | | | | | |
| --- | --- | --- | --- | --- | --- | --- | --- |
|  | RLS-KP | RLS-KS | RLS-avg | SLP-KP | SLP-KS | SLP-avg | NN |
| $\alpha$=0 | 52.7(1) | 57(5.9) | 52(0.6) | 59.3(0.1) | 82.6(<0.1) | 88.9(0.1) | 79.2(<0.1) |
| $\alpha$=0.1 | 52(1.5) | 57(5.9) | 63.3(0.3) | 56.6(0.1) | 83.9(<0.1) | 89.1(<0.1) | 79.1(<0.1) |
| $\alpha$=0.2 | 54.2(1.2) | 76.8(0.8) | 65.2(0.2) | 57.3(<0.1) | 84.9(<0.1) | 89.1(<0.1) | 79.1(0.1) |
| $\alpha$=0.3 | 55.4(1) | 82.8(0.1) | 71.5(0.2) | 58.9(0.2) | 85.6(<0.1) | 89.2(0.1) | 79.2(0.1) |
| $\alpha$=0.4 | 55.9(0.2) | 83.7(0.1) | 58.8(1.4) | 64(0.6) | 86.2(<0.1) | 89.2(<0.1) | 79.2(0.1) |
| $\alpha$=0.5 | 55.1(2.4) | 82.6(0.2) | 75.9(0.2) | 57.9(0.8) | 86.7(<0.1) | 89.2(<0.1) | 79.1(<0.1) |
| $\alpha$=0.6 | 56.8(1.3) | 52.3(5.8) | 75.3(0.2) | 64.4(0.1) | 87(<0.1) | 89.1(0.1) | 78.7(0.1) |
| $\alpha$=0.7 | 51.6(3) | 62.5(7.9) | 56.8(2.9) | 63.4(0.2) | 87.2(<0.1) | 89(0.1) | 78.5(0.1) |
| $\alpha$=0.8 | 50.3(0.4) | 65(4.7) | 72.3(0.6) | 59.3(0.1) | 87.2(<0.1) | 88.9(0.1) | 78.5(0.1) |
| $\alpha$=0.9 | 51.4(3.7) | 50(0.5) | 72.3(0.8) | 51.1(1.5) | 86.7(<0.1) | 88.5(<0.1) | 78.3(0.1) |
| $\alpha$=1 | 53(3.7) | 50.4(1.8) | 68.9(1.4) | 54(1) | 85(<0.1) | 87.5(0.1) | 77.8(0.1) |

ten-fold cross validation experiments 10 times. The AUC scores are normalized to 100. $\alpha$ indicates the weight coefficient of ATC feature covariant.
