# Supplementary material for: A Systematic Investigation of Computation Models for Predicting Adverse Drug Reactions (ADRs)
Source: PLoS One. 2014 Sep 2;9(9):e105889. doi: 10.1371/journal.pone.0105889 (PMC4152017; doi:10.1371/journal.pone.0105889)
Supplement: Table S4 — AUPR scores of models built with different intrinsic features. (DOC) [file pone.0105889.s004.doc]

**Table S4**.**AUPR scores of models built with different intrinsic features**

|  | AUPR | | | | | | |
| --- | --- | --- | --- | --- | --- | --- | --- |
|  | RLS-KP | RLS-KS | RLS-avg | SLP-KP | SLP-KS | SLP-avg | NN |
| $\alpha$=0 | 5.8(0.1) | 10.2(1.6) | 7.8(0.1) | 7.6(<0.1) | 25.7(<0.1) | 37.5(0.1) | 16.9(0.1) |
| $\alpha$=0.1 | 5.4(0.2) | 7.7(1.5) | 13.1(0.1) | 6.3(<0.1) | 26.7(<0.1) | 37.7(0.1) | 16.9(0.1) |
| $\alpha$=0.2 | 5.9(0.1) | 20.8(0.1) | 13.4(0.1) | 6.2(<0.1) | 27.4(<0.1) | 37.8(0.1) | 16.6(0.1) |
| $\alpha$=0.3 | 6.3(0.1) | 28.9(0.1) | 18.3(0.1) | 6.2(<0.1) | 28(0.1) | 37.9(0.1) | 16.1(0.1) |
| $\alpha$=0.4 | 6.2(<0.1) | 29.5(0.2) | 11.3(0.2) | 8.1(0.1) | 28.5(<0.1) | 37.9(0.1) | 15.1(0.1) |
| $\alpha$=0.5 | 6.2(0.4) | 27.4(0.2) | 21.9(0.2) | 6.4(0.1) | 29(<0.1) | 37.8(0.1) | 14.1(<0.1) |
| $\alpha$=0.6 | 6.9(0.2) | 9.3(1.3) | 20.3(0.1) | 8.2(<0.1) | 29.3(<0.1) | 37.6(0.1) | 13.7(0.1) |
| $\alpha$=0.7 | 6(0.5) | 13.6(1.4) | 11.1(0.3) | 7.7(0.1) | 29.4(<0.1) | 37.4(0.1) | 13.8(0.1) |
| $\alpha$=0.8 | 5(0.4) | 14.7(0.2) | 16.9(0.1) | 6.9(<0.1) | 29.2(<0.1) | 36.9(0.1) | 13.9(0.1) |
| $\alpha$=0.9 | 5.2(1.1) | 7.1(0.4) | 16(0.2) | 5.6(0.2) | 28.4(0.1) | 35.9(0.1) | 13.9(0.1) |
| $\alpha$=1 | 7.3(0.4) | 12.6(1.2) | 14.5(0.1) | 5.9(0.1) | 26.6(0.1) | 34.2(0.1) | 14.4(0.1) |

ten-fold cross validation experiments 10 times. The AUPR scores are normalized to 100. $\alpha$indicates the weight coefficient of ATC feature covariant.
