# Supplementary material for: A Systematic Investigation of Computation Models for Predicting Adverse Drug Reactions (ADRs)
Source: PLoS One. 2014 Sep 2;9(9):e105889. doi: 10.1371/journal.pone.0105889 (PMC4152017; doi:10.1371/journal.pone.0105889)
Supplement: Table S5 — AUC scores of models built with optimal integrated features. (DOC) [file pone.0105889.s005.doc]

**Table S5**.AUC scores of models built with optimal integrated features

|  | AUC | | | | | |  |
| --- | --- | --- | --- | --- | --- | --- | --- |
|  | $JC$ | $GK$ | $WRCN$ | $NP$ | $CN$ | $WLCN$ | $JCPN$ |
| RLS-KP | 91.4(0.1) | 92.9(0.1) | 90.5(0.1) | 91.9(<0.1) | 76.9(1.9) | 67.2(4.1) | 55.7(2.6) |
| RLS-KS | 92.5(0.1) | 93.5(<0.1) | 91.6(0.1) | 91.6(0.1) | 89.1(0.1) | 82.6(0.1) | 89.4(0.1) |
| RLS-avg | 93.3(<0.1) | 93(0.1) | 93.1(0.1) | 92.1(0.1) | 88.3(0.1) | 80.9(0.1) | 81.6(5.2) |
| SLP-KP | 90.1(<0.1) | 50(<0.1) | 88.9(0.1) | 50(<0.1) | 50(<0.1) | 50(<0.1) | 90.6(0.1) |
| SLP-KS | 92.4(<0.1) | 88.1(<0.1) | 91.7(0.1) | 88.1(<0.1) | 88.1(<0.1) | 88.1(<0.1) | 92.5(<0.1) |
| SLP-avg | 93.6(<0.1) | 90.9(<0.1) | 93.5(<0.1) | 92(<0.1) | 93(<0.1) | 92.8(<0.1) | 93.4(<0.1) |
| NN | 92.4(<0.1) | 79.7(0.1) | 91.3(<0.1) | 89.6(<0.1) | 89.9(<0.1) | 89.6(<0.1) | 91.7(0.2) |
| GWPM | 93.0(0.1) | 90.7(<0.1) | 93.5(<0.1) | 91.2(<0.1) | 93.1(<0.1) | 93.0(0.1) | 94.2(0.1) |

ten-fold cross validation experiments 10 times. The AUC scores are normalized to 100.
