# Supplementary material for: A Systematic Investigation of Computation Models for Predicting Adverse Drug Reactions (ADRs)
Source: PLoS One. 2014 Sep 2;9(9):e105889. doi: 10.1371/journal.pone.0105889 (PMC4152017; doi:10.1371/journal.pone.0105889)
Supplement: Table S6 — AUPR scores of models built with optimal integrated features. (DOC) [file pone.0105889.s006.doc]

**Table S6**.AUPR scores of models built with optimal integrated features

|  | AUPR | | | | | |  |
| --- | --- | --- | --- | --- | --- | --- | --- |
|  | $JC$ | $GK$ | $WRCN$ | $NP$ | $CN$ | $WLCN$ | $JCPN$ |
| RLS-KP | 63.8(0.1) | 62.2(0.2) | 52(0.2) | 54(0.1) | 21.2(1.1) | 10.7(2.2) | 6.3(0.4) |
| RLS-KS | 64.0(0.1) | 60.9(0.2) | 57.4(0.2) | 54.1(0.1) | 51.1(0.2) | 32(0.3) | 51.3(0.1) |
| RLS-avg | 63.5(0.1) | 60.4(0.2) | 61.2(0.2) | 54.3(0.1) | 48.2(0.4) | 26.2(0.3) | 48.3(0.9) |
| SLP-KP | 45.5(0.2) | 52.5(<0.1) | 53.4(<0.1) | 52.5(<0.1) | 52.5(<0.1) | 52.5(<0.1) | 47.5(1.8) |
| SLP-KS | 55.5(0.1) | 30.5(<0.1) | 55.6(0.1) | 30.5(<0.1) | 30.5(<0.1) | 30.5(<0.1) | 56.0(0.1) |
| SLP-avg | 57.6(0.1) | 38.8(0.1) | 57.4(0.1) | 53.5(0.1) | 56.1(<0.1) | 55.9(0.1) | 58.1(0.1) |
| NN | 53.3(0.2) | 14.8(0.1) | 43.3(0.1) | 37.8(0.1) | 40.3(0.1) | 39.7(0.2) | 52.8(0.4) |
| GWPM | 60.0(<0.1) | 38.7(0.1) | 59.5(0.1) | 53.3(0.1) | 58.2().1) | 57.5(0.1) | 65.7(0.2) |

ten-fold cross validation experiments 10 times. The AUPR scores are normalized to 100.
