# Supplementary material for: A Systematic Investigation of Computation Models for Predicting Adverse Drug Reactions (ADRs)
Source: PLoS One. 2014 Sep 2;9(9):e105889. doi: 10.1371/journal.pone.0105889 (PMC4152017; doi:10.1371/journal.pone.0105889)
Supplement: Table S7 — Drug features weight coefficients of models built with optimal integrated features. (DOC) [file pone.0105889.s007.doc]

**Table S7.Drug features weight coefficients** of models built with optimal integrated features

|  | $\alpha$ | | | | | |  |
| --- | --- | --- | --- | --- | --- | --- | --- |
|  | $JC$ | $GK$ | $WRCN$ | $NP$ | $CN$ | $WLCN$ | $JCPN$ |
| RLS-KP | 0.8 | 0.9 | 0.9 | 0.5 | 0.7 | 0.4 | 0 |
| RLS-KS | 0.8 | 0.9 | 0.3 | 0.9 | 0.1 | 0 | 0.2 |
| RLS-avg | 0.9 | 0.9 | 0.8 | 0.9 | 0.1 | 0.1 | 0.1 |
| SLP-KP | 1 | 1 | 1 | 1 | 1 | 1 | 1 |
| SLP-KS | 1 | 0.9 | 1 | 0.9 | 0.9 | 0.9 | 1 |
| SLP-avg | 0.9 | 0.1 | 0.9 | 0.3 | 0.5 | 0 | 0.9 |
| NN | 0.7 | 0 | 0.5 | 0.8 | 0.8 | 0.5 | 0.7 |
| GWPM | 0.1 | 0 | 0.9 | 0.7 | 0.9 | 0.7 | 0.8 |

Here, $\alpha$indicates the weight coefficient of drug topological feature.
