# Supplementary material for: A Systematic Investigation of Computation Models for Predicting Adverse Drug Reactions (ADRs)
Source: PLoS One. 2014 Sep 2;9(9):e105889. doi: 10.1371/journal.pone.0105889 (PMC4152017; doi:10.1371/journal.pone.0105889)
Supplement: File S1 — Supplementary Algorithms. More detailed descriptions regarding algorithms have been provided in this file. (DOC) [file pone.0105889.s009.doc]

**Supplementary Algorithms**

1. **RLS**

To make it more convenient for later description, the set of prediction scores for each drug-ADR pair were characterized as an $n_{d}\times n_{a}$ matrix$\widehat{Y}$, where the element $[\widehat{Y}]_{ij}$ represents the prediction score of drug-ADR pair$(d_{i},a_{j})$ and the larger $[\widehat{Y}]_{ij}$ is, the more likely there exists an association between drug$d_{i}$and ADR$a_{j}$. The set of similarity scores between drugs and between ADRs were characterized as an $n_{d}\times n_{d}$ similarity matrix $S_{d}$ and an $n_{a}\times n_{a}$ similarity matrix$S_{a}$, respectively. The elements $[S_{d}]_{ij}$ and $[S_{a}]_{ij}$ represent the similarities of drug-drug pairs$(d_{i},d_{j})$ and ADR-ADR pairs$(a_{i},a_{j})$, respectively.

Now, we review the formulation of RLS. Based on RLS,$\widehat{Y}=Kc$, where, $K$ is a kernel matrix and $c$is a coefficient vector that must be optimized. Here, $K$can be defined in different ways: $S_{d}$and$S_{a}$ can represent $K$ directly, or $S_{d}$and $S_{a}$ are first transformed and the transformed result is used to represent $K$. (It should be noted that, strictly, the kernel matrix $K$ needs to meet two necessary and sufficient conditions that are symmetric and positive semi-definite, whereas $S_{d}$and$S_{a}$only need to meet the symmetry condition. Therefore, if $S_{d}$or$S_{a}$ doesn’t meet the positive semi-definite condition, we added a small multiple of the identity matrix to their diagonal until the positive semi-definite condition is met, these additions resulted in minor modifications to the original $S_{d}$or$S_{a}$.)Depending on the method for defining $K$, the RLS algorithm can be divided into three separate sub algorithms.

**1.1 RLS-KP**

Based on the RLS-KP algorithm,$vec(\widehat{Y})=Kc$, where $vec(\widehat{Y})$ is the vectorization operation of matrix $\widehat{Y}$; $K$is defined as $K=S_{d}\otimes S_{a}$, where the operator indicates the Kronecker Product; and the coefficient $c$is obtained by minimizing the following objective function:

$\min_{c\in R^{l}} \frac{1}{2l}\sum_{i=1}^{l} (vec(Y^{T})-Kc)^{T}(vec(Y^{T})-Kc)+\frac{\lambda}{2}c^{T}Kc$, where $l=n_{d}\times n_{a}$and$\lambda$is a regularization parameter.

The above function is operated by taking the first derivative of$c$to obtain the linear equation:$c=(K+\sigma I)^{-1}vec(Y^{T})$, where $\sigma=\lambda l$ and $I$ is a $n_{d}n_{a}\times n_{d}n_{a}$ unit matrix. Hence, $vec(\widehat{Y}^{T})=Kc=K(K+\sigma I)^{-1}vec(Y^{T})$, and in theory, $\widehat{Y}$ can be directly calculated using the equation: $vec(\widehat{Y}^{T})=K(K+\sigma I)^{-1}vec(Y^{T})$. However, because the kernel matrix $K$ is a $n_{d}n_{a}\times n_{d}n_{a}$ matrix, huge memory overhead will be necessary for practice calculation. Therefore, several mathematical transformation techniques based on the Kronecker product are employed to reduce the computational cost [11]. We here omit the detailed process of strict derivation and present the final result as follows:

$\widehat{Y}=V_{d}Z^{T}V_{a}^{T}$;

where$vec(Z)=(\Lambda_{d}\otimes\Lambda_{a})(\Lambda_{d}\otimes\Lambda_{a}+\sigma I)^{-1}vec(V_{a}^{T}Y^{T}V_{d})$; $S_{d}=V_{d}\Lambda_{d}V_{d}^{T}$and$S_{a}=V_{a}\Lambda_{a}V_{a}^{T}$are the Eigen decompositions of these two similarity matrices.

**1.2 RLS-KS**

The formal derivation of RLS-KS is similar to RLS-KP, with the only difference being the different definitions of the kernel matrix$K$ based on RLS-KS,$K=S_{d}\oplus S_{a}$, where the operator indicates the Kronecker sum. Here, the final result is given directly as:

$\widehat{Y}=V_{d}Z^{T}V_{a}^{T}$;

Where$vec(Z)=(\Lambda_{d}\oplus\Lambda_{a})(\Lambda_{d}\oplus\Lambda_{a}+\sigma I)^{-1}vec(V_{a}^{T}Y^{T}V_{d})$; $S_{d}=V_{d}\Lambda_{d}V_{d}^{T}$and$S_{a}=V_{a}\Lambda_{a}V_{a}^{T}$are the Eigen decompositions of these two similarity matrices.

**1.3 RLS-avg**

RLS-avg is different from RLS-KP (RLS-KS) in that RLS-avg constructs two independent models based on $S_{d}$ and$S_{a}$, respectively. The average of the prediction score matrices of these two models is then taken as the final prediction score matrix$\widehat{Y}$.

We illustrate the computational process of RLS-avg based on drugs. Based on RLS-avg, $\widehat{Y}_{d}=K_{d}C_{d}$, where $K_{d}=S_{d}$, and $C_{d}$is optimized by the following objective function:

$\min_{C_{d}} \frac{1}{2l}\left\|Y-K_{d}C_{d}\right\|_{F}^{2}+\frac{\lambda}{2}C_{d}^{T}K_{d}C_{d}$;

where $l=n_{d}\times n_{a}$,$\lambda$ is a regularization parameter. The above function is operated by taking the first derivative of$C_{d}$ to obtain the linear equation is obtained:$C_{d}=(K_{d}+\sigma I)^{-1}Y$, where$\sigma=\lambda l$, $I$ is a $n_{d}\times n_{d}$unit matrix, and $\widehat{Y}_{d}=K_{d}C_{d}=K_{d}(K_{d}+\sigma I)^{-1}Y$. Similarly, because $\widehat{Y}_{a}=K_{a}C_{a}=K_{a}(K_{a}+\sigma I)^{-1}Y^{T}$, the final prediction score matrix is $\widehat{Y}=(\widehat{Y}_{d}+\widehat{Y}_{a}^{T})/2$.

In addition, the regularization parameter$\sigma$ is contained in each RLS algorithms.The optimal value of$\sigma$ should be selected by the grid search with cross validation; considering the complexity of this calculation, we used the recommended value of the regularization parameter $\sigma$=1.

1. **SLP**

The basic assumption of SLP is that “Two node pairs that are similar to each other are likely to have the same link strength”. Based on this assumption, the objective function is determined as:

$\min_{\widehat{Y}} \frac{\sigma}{2}\widehat{Y}^{T}L\widehat{Y}+\frac{1}{2}\left\|\widehat{Y}-Y\right\|_{F}^{2}$;

where $\sigma$ is a regularization parameter and $L$ is a Laplacian matrix. SLP can be divided into three independent sub algorithms based on the definition of$L$.

**2.1 SLP-KP**

The Laplacian matrix of SLP-KP is defined as: $L=I-(D_{a}^{-1/2}S_{a}D_{a}^{-1/2})\otimes(D_{d}^{-1/2}S_{d}D_{d}^{-1/2})$; where $I$ is a $n_{d}n_{a}\times n_{d}n_{a}$ unit matrix; $D_{d}$and $D_{a}$ are both diagonal matrices, where $\left[D_{d}\right]_{ii}=\sum_{j=1}^{n_{d}} [S_{d}]_{ij}$, $\left[D_{a}\right]_{ii}=\sum_{j=1}^{n_{a}} [S_{a}]_{ij}$. In this case, the objective function of SLP is transformed as: $\min_{\widehat{Y}} \frac{\sigma}{2}vec(\widehat{Y}^{T})Lvec(\widehat{Y})+\frac{1}{2}\left\|vec(\widehat{Y})-vec(Y)\right\|_{F}^{2}$; The above function is operated by taking the first derivative of $vec(\widehat{Y})$to obtain the linear equation: $vec(\widehat{Y})=(\sigma L+I)^{-1}vec(Y)$.

The subsequent mathematical techniques of SLP-KP are similar to RLS-KP to solve $\widehat{Y}$ as:$\widehat{Y}=V_{d}(D(V_{d}^{T}YV_{a}))V_{a}^{T}$; where $\left[D\right]_{ij}=(1+\sigma(1-\left[\Lambda_{d}\right]_{ii}\left[\Lambda_{a}\right]_{jj}))^{-1}$,$S_{d}=V_{d}\Lambda_{d}V_{d}^{T}$and$S_{a}=V_{a}\Lambda_{a}V_{a}^{T}$are the Eigen decompositions of these two similarity matrices.

**2.2 SLP-KS**

The Laplacian matrix of SLP-KS is defined as: $L=3I-(D_{a}^{-1/2}S_{a}D_{a}^{-1/2})\oplus(D_{d}^{-1/2}S_{d}D_{d}^{-1/2})$.The object function and computational processing of SLP-KS are similar to SLP-KP to yield the final result of SLP-KS:

$\widehat{Y}=V_{d}(D(V_{d}^{T}YV_{a}))V_{a}^{T}$;

where$\left[D\right]_{ij}=(1+\sigma(3-\left[\Lambda_{d}\right]_{ii}-\left[\Lambda_{a}\right]_{jj}))^{-1}$,$S_{d}=V_{d}\Lambda_{d}V_{d}^{T}$and$S_{a}=V_{a}\Lambda_{a}V_{a}^{T}$are the Eigen decompositions of these two similarity matrices.

**2.3 SLP-avg**

The overall handling of SLP-avg is similar to RLS-avg, in which SLP-avg constructs two independent models based on $S_{d}$ and$S_{a}$, respectively. The average of the prediction score matrices of these two models is then taken as the final prediction score matrix$\widehat{Y}$.

We illustrate the computational process of SLP-avg based on drugs. The Laplacian matrix of SLP-avg is defined as: $L_{d}=I-D_{d}^{-1/2}S_{d}D_{d}^{-1/2}$, where $I$ is a $n_{d}\times n_{d}$ unit matrix, and the object function of SLP-avg is $\min_{\widehat{Y}} \frac{\sigma}{2}\widehat{Y}_{d}^{T}L\widehat{Y}_{d}+\frac{1}{2}\left\|\widehat{Y}_{d}-Y\right\|_{F}^{2}$. This object function is operated by taking the first derivative of $\widehat{Y}_{d}$ to solve $\widehat{Y}_{d}$ as: $\widehat{Y}_{d}=(\sigma L_{d}+I)^{-1}Y$. Analogously, based on ADRs, the solution of $\widehat{Y}_{a}$ is $\widehat{Y}_{a}=(\sigma L_{a}+I)^{-1}Y^{T}$, where $L_{a}=I-D_{a}^{-1/2}S_{a}D_{a}^{-1/2}$. Therefore the final prediction score matrix is represented as:$S=S_{d}(S_{d}+\sigma I_{n_{d}})^{-1}$.

In addition, the regularization parameter $\sigma$ for each SLP sub algorithms was selected to be the same value: $\sigma$=0.01.

1. **NN**

The overall handling of NN is similar to RLS-avg or SLP-avg, which constructs two independent models based on $S_{d}$and$S_{a}$, respectively. The final prediction score matrix$\widehat{Y}$ integrates the prediction score matrices of these two models. We illustrate the computational process of NN based on drugs. If there is no known association between drug$d_{i}$and ADR$a_{j}$, then $\left[Y\right]_{ij}=0$. The drugs neighbor set of $a_{j}$ (denoted$\Gamma(a_{j})$) is obtained from the drug-ADR association network, and the prediction score of drug-ADR pair $(d_{i},a_{j})$ is defined as:$\left[\widehat{Y}_{d}\right]_{ij}=\max(\left[S_{d}\right]_{ik},d_{k}\in\Gamma(a_{j}))$. Analogously, based on ADRs, the prediction score of $(d_{i},a_{j})$ is defined as:$\left[\widehat{Y}_{a}\right]_{ji}=\max(\left[S_{a}\right]_{jk},a_{k}\in\Gamma(d_{i}))$. The final prediction score of $(d_{i},a_{j})$ is $[\widehat{Y}]_{ij}=\max([\widehat{Y}_{d}]_{ij},[\widehat{Y}_{a}]_{ji})$.

**4. GWPM**

The basic formula of GWPM is as follows: $\widehat{Y}=(\widetilde{S_{d}}\cdot Y+(\widetilde{S_{a}}\cdot Y^{T})^{T})/2$, where $\widetilde{S_{d}}(\widetilde{S_{a}})$ can be considered as a function of $S_{d}(S_{a})$. The computational process of $\widetilde{S_{d}}$ is simple: first, the elements in $\widetilde{S_{d}}$ are calculated by the formula $[\widetilde{S_{d}}]_{ij}=[S_{d}]_{ij}^{n}/\sum_{k=1}^{n_{d}} [S_{d}]_{ik}^{n}$; then, $\widetilde{S_{d}}$ is symmetrized by $\widetilde{S_{d}}=(\widetilde{S_{d}}+\widetilde{S_{d}}^{T})/2$. The computational process of $\widetilde{S_{a}}$ is similar to$\widetilde{S_{d}}$. In addition, there is an index parameter $n$ in GWPM. According to the results from the modeling experiments, this parameter was set as 3,1,3,2,3,2, and 3 for $JC$,$GK$,$WRCN$,$NP$,$CN$,$WLCN$and $JCPN$, respectively.
