# Supplementary material for: A Systematic Investigation of Computation Models for Predicting Adverse Drug Reactions (ADRs)
Source: PLoS One. 2014 Sep 2;9(9):e105889. doi: 10.1371/journal.pone.0105889 (PMC4152017; doi:10.1371/journal.pone.0105889)
Supplement: File S2 — Supplementary Algorithm Analysis. More detailed descriptions of the unified formulas of algorithms have been provided in this file. (DOC) [file pone.0105889.s010.doc]

**Supplementary Algorithm Analysis**

By comparing all sub algorithms, the formulas for RLS-KP, RLS-KS, SLP-KP, SLP-KS and NN are unified as $vec(\widehat{Y})=S\cdot vec(Y)$ or $vec(\widehat{Y}^{T})=S\cdot vec(Y^{T})$, and the formulas of RLS-avg and SLP-avg are unified as $\widehat{Y}=S\cdot Y$.

For RLS-KP, $S=(S_{d}\otimes S_{a})(S_{d}\otimes S_{a}+\sigma I_{n_{d}\times n_{a}})^{-1}$.

For RLS-KS, $S=(S_{d}\oplus S_{a})(S_{d}\oplus S_{a}+\sigma I_{n_{d}\times n_{a}})^{-1}$.

For SLP-KP, $S=[(\sigma+1)I-\sigma(D_{a}^{-1/2}S_{a}D_{a}^{-1/2})\otimes(D_{d}^{-1/2}S_{d}D_{d}^{-1/2})]^{-1}$.

For SLP-KS, $S=[(\sigma+1)I-\sigma(D_{a}^{-1/2}S_{a}D_{a}^{-1/2})\oplus(D_{d}^{-1/2}S_{d}D_{d}^{-1/2})]^{-1}$.

For NN, $[\widehat{Y}]_{ij}=\max([\widehat{Y}_{d}]_{ij},[\widehat{Y}_{a}]_{ji})$, where $\widehat{Y}=\max(\widehat{Y}_{d},\widehat{Y}_{a}^{T})$. $\widehat{Y}_{d}$and $\widehat{Y}_{a}$ are unified as: $vec(\widehat{Y})=S\cdot vec(Y)$. The formula of S of $\widehat{Y}_{d}$ is as follows:

$S=\left[\begin{matrix} K_{1} & & & & \\ & K_{2} & & & \\ & & \bullet& & \\ & & & \bullet& \\ & & & & K_{n_{a}} \end{matrix}\right]_{n_{d}n_{a}\times n_{d}n_{a}}$, where, $[K_{m}]_{ij}=\sigma_{it}\cdot[S_{d}]_{ij}$,$t\in\{l|[S_{d}]_{il}=\max([S_{d}]_{il},d_{l}\in\Gamma(a_{m}))\}$, $\sigma_{it}$ is the Kronecker function, if i=t, $\sigma_{it}$=1; otherwise $\sigma_{it}$=0. The formula of S of $\widehat{Y}_{a}$ is similar to $\widehat{Y}_{d}$.

For RLS-avg, $\widehat{Y}=(\widehat{Y}_{d}+\widehat{Y}_{a}^{T})/2$, $\widehat{Y}_{d}$and $\widehat{Y}_{a}$ could both be unified as: $\widehat{Y}=S\cdot Y$. The formula of S of $\widehat{Y}_{d}$ is as follows: $S=S_{d}(S_{d}+\sigma I_{n_{d}})^{-1}$. The formula of S of $\widehat{Y}_{a}$ is similar to $\widehat{Y}_{d}$.

For SLP-avg, $\widehat{Y}=(\widehat{Y}_{d}+\widehat{Y}_{a}^{T})/2$, $\widehat{Y}_{d}$and $\widehat{Y}_{a}$ are unified as: $\widehat{Y}=S\cdot Y$. The formula of S of $\widehat{Y}_{d}$ is as follows: $S=[(\sigma+1)I_{n_{d}}-(D_{d}^{-1/2}S_{d}D_{d}^{-1/2})]^{-1}$. The formula of S of $\widehat{Y}_{a}$ is similar to $\widehat{Y}_{d}$.

In addition, $\widehat{Y}=S\cdot Y$ is a special form of $vec(\widehat{Y})=S\cdot vec(Y)$. $\widehat{Y}=S\cdot Y$ is equivalent to $vec(\widehat{Y})=\widetilde{S}\cdot vec(Y)$, where $\widetilde{S}=\left[\begin{matrix} S_{d} & & & & \\ & S_{d} & & & \\ & & \bullet& & \\ & & & \bullet& \\ & & & & S_{d} \end{matrix}\right]_{n_{d}n_{a}\times n_{d}n_{a}}$. Hence, in general, the above sub algorithms have the unified formula $vec(\widehat{Y})=S\cdot vec(Y)$.
