# Supplementary material for: A Systematic Investigation of Computation Models for Predicting Adverse Drug Reactions (ADRs)
Source: PLoS One. 2014 Sep 2;9(9):e105889. doi: 10.1371/journal.pone.0105889 (PMC4152017; doi:10.1371/journal.pone.0105889)
Supplement: File S3 — MATLAB source code. This file includes MATLAB resource code to implement computational experiments in this paper. (PDF) [file pone.0105889.s011.pdf]

MATLAB source code

%These codes have been tested in matlab2012a(win7 OS)

projectNetworkSimilarity.m

```
%This code is used to calculate topological features of network;
%Sd=projectNetworkSimilarity(MatDA,optionStr);
%InPut:
% MatDA - drug-ADR association adjacent matrix;
% optionStr - character string to select different kinds of topological features to
%calculate;based on different character string, this code could calculate 11 kinds of
% topological features,for example:
% optionStr='neighbourCommon' - to calculate Common Neighbors;
% optionStr='neighbourProduct' - to calculate Neighbors Product;
% optionStr='neighbourCommonWeightLog' - to calculate WLCN;
%optionStr='neighbourCommonWeightRev' - to calculate WRCN;
%optionStr='Jaccard' - to calculate Jaccard coefficient;
%optionStr='GaussianKernel' - to calculate Gaussian interaction profile kernel;
%optionStr=’JaccardPvsN’- to calculate JCPN;
%OutPut:
%Sd - if row ID of MatDA correspond to drugs, Sd is the topological similarity matrix
% of drugs; otherwise, Sd is the topological similarity matrix of ADRs;
function Sd=projectNetworkSimilarity(MatDA,optionStr)
        if strcmp(optionStr,'neighbourCommon')
            Sd=MatDA*MatDA';
        end

        if strcmp(optionStr,'neighbourProduct')
            nD=size(MatDA,1);
            degreeD=sum(MatDA,2);
            mat1=repmat(degreeD,1,nD);
            mat2=repmat(degreeD',nD,1);
            MatdProduct=mat1.*mat2;
            Sd=MatdProduct;
        end

        if strcmp(optionStr,'neighbourCommonWeightLog')
           [nD,nA]=size(MatDA);
            degreeA=sum(MatDA);
            logDegreeA=log(degreeA);
            logDegreeA(logDegreeA==Inf)=0;
            MatdShareW=zeros(nD,nD);
            for I=1:nA
```

```
            v=logical(MatDA(:,I));
            MatdShareW(v,v)=MatdShareW(v,v)+logDegreeA(I);
        end
        Sd=MatdShareW;
end

if strcmp(optionStr,'neighbourCommonWeightRev')
      [nD,nA]=size(MatDA);
        degreeA=sum(MatDA);
        revDegreeA=1./(degreeA);
        revDegreeA(revDegreeA==Inf)=0;
        MatdShareR=zeros(nD,nD);
        for I=1:nA
            v=logical(MatDA(:,I));
            MatdShareR(v,v)=MatdShareR(v,v)+revDegreeA(I);
        end
        Sd=MatdShareR;
end


if strcmp(optionStr,'Jaccard')
      nD=size(MatDA,1);
      MatdShare=MatDA*MatDA';
      MatdNeighbour=diag(MatdShare);
      MatdCombine=repmat(MatdNeighbour,1,nD)+...
      repmat(MatdNeighbour',nD,1)-MatdShare;
      Sd=MatdShare./MatdCombine;
      %Sd=Sd.^3;
      vnan=isnan(Sd);
      Sd(vnan)=0;
end

if strcmp(optionStr,'JaccardPvsN')
      nD=size(MatDA,1);
      MatdShare=MatDA*MatDA';

      MatdNeighbour=diag(MatdShare);
      MatdCombine=repmat(MatdNeighbour,1,nD)+...
      repmat(MatdNeighbour',nD,1)-MatdShare;
      Sd1=MatdShare./(MatdCombine-MatdShare);
      Sd1(Sd1==Inf)=0;
      Sd1(isnan(Sd1))=0;
      Sd=Sd1;
end
```

```
        if strcmp(optionStr,'GaussianKernel')
            M=MatDA*MatDA';
            mDiag=diag(M);
            RmDiag=repmat(mDiag',size(MatDA,1),1);
            CmDiag=repmat(mDiag,1,size(MatDA,1));
            K2=RmDiag-2*M+CmDiag;
            dWid=sum(sum(MatDA.^2,2))/size(MatDA,1);
            beta=1;
            K2=exp(-(beta/dWid)*K2);
            Sd=K2;
        end
%%%
```

ROCcompute.m

```
%This code is used to obtain AUC and AUPR;
%[AUC,AUPR,Acc,Sen,Spe,Pre]=ROCcompute(predictList,trueList,plotOption);
%InPut:
% predictList - prediction score matrix or vector by each models;
% trueList - sign matrix or vector to record whehter or not existing association
% corresponding to each drug-ADR pair in predictList;
% plotOption - to choose whether ploting ROC or not, if plotOption=1, ploting ROC;
%OutPut:
% (omit)
Function    [AUC,AUPR,Acc,Sen,Spe,Pre]=ROCcompute(predictList,trueList, …
plotOption)
        if size(predictList,2)>1
            predictList=reshape(predictList,numel(predictList),1);
            trueList=reshape(trueList,numel(trueList),1);
        end

        low=min(predictList);
        high=max(predictList);
        threshold=linspace(high,low,100);

        Sen=zeros(1,100); Spe=zeros(1,100); Pre=zeros(1,100);
        Acc=zeros(1,100);
        for I=1:100
            Vector=zeros(numel(predictList),1);
            v=predictList>=threshold(I);
            Vector(v)=1;

            tp=sum(Vector==1&trueList==1);
            tn=sum(Vector==0&trueList==0);
```

```
        np=sum(Vector==1&trueList==0);
        nn=sum(Vector==0&trueList==1);

        Sen(I)=tp/(tp+nn);

        Spe(I)=tn/(tn+np);

       if tp+np==0
           Pre(I)=1;
       else
          Pre(I)=tp/(tp+np);
       end

       Acc(I)=(tn+tp)/(tn+tp+np+nn);
    end

    Sen=[0,Sen];Spe=[1,Spe];Pre=[1,Pre];
    Acc=[sum(trueList==0)/length(trueList) Acc];

    AUC=abs(trapz(1-Spe,Sen));
    AUPR=abs(trapz(Sen,Pre));

    if plotOption==1
       plot(1-Spe,Sen);
       axis([-0.01 1.00 0 1.01]);
       xlabel('1-Spe');
       ylabel('Sen');

       figure;
       plot(Sen,Pre);
       axis([0 1.01 0 1.01]);
       xlabel('Sen');
       ylabel('Pre');
    end
%%%
```

RLS_Kron.m

```
%This code is used to implement RLS-KP or RLS-KS;
%matPredict=RLS_Kron(Kd,Kt,delta,matOrig,modelOption);
%InPut:
% Kd - similarity matrix of drugs;
% Kt - similarity matrix of ADRs;
%delta - parameter in RLS; in this paper, delta=1;
%matOrig - drug-ADR association adjacent matrix, row ID of matOrig correspond to
```

```
% drugs;
% modelOption - character string to select implementing RLS-KP or RLS-KS;
% if modelOption='KronProduct',then implementing RLS-KP;
% if modelOption='KronSum', then implementing RLS-KS;
%OutPut:
% matPredict - prediction score matrix by RLS-KP or RLS-KS;
function matPredict=RLS_Kron(Kd,Kt,delta,matOrig,modelOption)
        [Ud,ed]=eig(Kd);
        [Ut,et]=eig(Kt);

        z=reshape(Ut'*matOrig'*Ud,numel(Ut'*matOrig'*Ud),1);

        Ed=diag(ed);
        Ed=repmat(Ed',size(et,1),1);
        Ed=reshape(Ed,numel(Ed),1);
        Et=diag(et);
        Et=repmat(Et,size(ed,1),1);

        if strcmp(modelOption,'KronProduct')
           Edt=Ed.*Et;
        end
        if strcmp(modelOption,'KronSum')
           Edt=Ed+Et;
        end

        Z=(Edt./(Edt+delta)).*z;
        Z=reshape(Z,size(Kt,1),size(Kd,1));

        matPredict=Ud*Z'*Ut';
%%%
```

RLS_avg.m

```
%This code is used to implement RLS-avg;
%matPredict=RLS_avg(Kd,Kt,delta,matOrig)
%InPut:
%each input variable is similar to RLS_Kron.m
%OutPut:
%each output variable is similar to RLS_Kron.m
function matPredict=RLS_avg(Kd,Kt,delta,matOrig)
        matPredictD=Kd/(Kd+delta*eye(size(Kd,1)))*matOrig;
        matPredictT=Kt/(Kt+delta*eye(size(Kt,1)))*matOrig';
        matPredict=0.5*matPredictD+0.5*matPredictT';
%%%
```

semi_supervisedModel3.m

```
%This code is used to implement SLP-KP,SLP-KS or SLP-avg;
%F=semi_supervisedModel3(MatTD,Wt,Wd,alph,Option)
%InPut:
%MatTD - drug-ADR association adjacent matrix, row ID of matOrig correspond to ADRs;
%Wt - similarity matrix of ADRs;
%Wd - similarity matrix of drugs;
% alph - parameter in SLP; in this paper, alph=0.01;
%Option - integer number to select implementing SLP-KP,SLP-KS or SLP-avg;
%OutPut:
%F - prediction score matrix by SLP-KS,SLP-KS or SLP-avg;
function F=semi_supervisedModel3(MatTD,Wt,Wd,alph,Option)
        [nT,nD]=size(MatTD);

        [Pt,Et]=eig(Wt); Et=diag(Et);
        [Pd,Ed]=eig(Wd); Ed=diag(Ed);

    if Option==1
      D=reshape(repmat(Ed',nT,1),nT*nD,1).*reshape(repmat(Et,nD,1),nT*nD,1);
       D=(1+alph)-alph*D;
    end

    if Option==2
        D=reshape(repmat(Ed',nT,1),nT*nD,1)+reshape(repmat(Et,nD,1),nT*nD,1);
        D=(1+3*alph)-alph*D;
    end

    if Option==1||Option==2
        F=reshape(Pt'*MatTD*Pd,nT*nD,1);
        F=(1./D).*F;
        F=Pt*reshape(F,nT,nD)*Pd';
    end

    if Option==3
        MatDT=MatTD';
        Lt=eye(nT)-Wt;
        Ft=(eye(nT)+alph*Lt)\MatTD;
        Ld=eye(nD)-Wd;
        Fd=(eye(nD)+alph*Ld)\MatDT;

        F=(Ft+Fd')/2;
    end
%%%
```

simBaseModel.m

```
%This code is used to implement NN;
%matPredict=simBaseModel(matDA,Sd);
%InPut:
%matDA - drug-ADR association adjacent matrix;
% Sd - if row ID of matDA correspond to drugs, Sd is similarity matrix of drugs;
%otherwise, Sd is similarity matrix of ADRs;
%OutPut:
% matPredict - prediction score matrix by NN;
function matPredict=simBaseModel(matDA,Sd)
        [nD,nA]=size(matDA);
        matPredict=zeros(nD,nA);
         for I=1:nA
            v=matDA(:,I);
            if sum(v)>0
               v=logical(v);
               subSd=Sd(:,v);
               matPredict(:,I)=max(subSd,[],2);
            else
                matPredict(:,I)=0;
            end
         end
%%%
```

RLSmodelCVtest.m

```
%This code is used to evaluate the performance of models built by RLS based on 10
%folds cross validation;
function [AUC,AUPR,Acc,Sen,Spe,Pre,MatPredict]=RLSmodelCVtest(MatDA,K, …
Sd,Sa,toplogyOption,weightOption,weightCoef,delta,Option,plotOption)
[nD,nA]=size(MatDA);
Vp=find(MatDA==1);
Vn=find(MatDA==0);
Ip=crossvalind('Kfold',numel(Vp),K);
In=crossvalind('Kfold',numel(Vn),K);

MatPredict=zeros(nD,nA);
for I=1:K
    vp=Ip==I;
    vn=In==I;
    Mat=MatDA;
    Mat([Vp(vp);Vn(vn)])=0;
    Kd=projectNetworkSimilarity(Mat,toplogyOption);
    Ka=projectNetworkSimilarity(Mat',toplogyOption);
```

```
        if weightOption==1
              SD=weightCoef(1)*Sd+(1-weightCoef(1))*Kd;
              SA=weightCoef(2)*Sa+(1-weightCoef(2))*Ka;
        end
        if weightOption==2
                SD=Sd.^weightCoef(1).*Kd.^(1-weightCoef(1));
                SA=Sa.^weightCoef(2).*Ka.^(1-weightCoef(2));
        end

        if Option==1
              matPredict=RLS_Kron(SD,SA,delta,Mat,'KronProduct');
        end
          if Option==2
              matPredict=RLS_Kron(SD,SA,delta,Mat,'KronSum');
        end
        if Option==3
              matPredict=RLS_avg(SD,SA,delta,Mat);
        end
        MatPredict([Vp(vp);Vn(vn)])=matPredict([Vp(vp);Vn(vn)]);
end
[AUC,AUPR,Acc,Sen,Spe,Pre]=ROCcompute(MatPredict,MatDA,plotOption);
%%%
```

RLSmodelPredict.m

```
%This code is used to evaluate the performance of models built by RLS based on
% prospective evaluation;
function [AUC,AUPR,Acc,Sen,Spe,Pre,MatPredict]=RLSmodelPredict(MatDA1,…
MatDA2,Sd,Sa,toplogyOption,weightOption,weightCoef,delta,Option,plotOption)
        Kd=projectNetworkSimilarity(MatDA1,toplogyOption);
        Ka=projectNetworkSimilarity(MatDA1',toplogyOption);
        if weightOption==1
              Sd=weightCoef(1)*Sd+(1-weightCoef(1))*Kd;
              Sa=weightCoef(2)*Sa+(1-weightCoef(2))*Ka;
        end
        if weightOption==2
                Sd=Sd.^weightCoef(1).*Kd.^(1-weightCoef(1));
                Sa=Sa.^weightCoef(2).*Ka.^(1-weightCoef(2));
        end

        if Option==1
              MatPredict=RLS_Kron(Sd,Sa,delta,MatDA1,'KronProduct');
        end
        if Option==2
              MatPredict=RLS_Kron(Sd,Sa,delta,MatDA1,'KronSum');
```

```
        end
        if Option==3
              MatPredict=RLS_avg(Sd,Sa,delta,MatDA1);
        end

          v=MatDA1==0;
          PredictV=MatPredict(v);
          TrueV=MatDA2(v);
          [AUC,AUPR,Acc,Sen,Spe,Pre]=ROCcompute(PredictV,TrueV,plotOption);
%%%
```

SemiSupervisedModelCVtest.m

```
%This code is used to evaluate the performance of models built by SLP based on 10
% folds cross validation;
function [AUC,AUPR,Acc,Sen,Spe,Pre,MatPredict]=SemiSupervisedModelCVtest ...
(MatDA,K,Sd,Sa,toplogyOption,weightOption,weightCoef,alph,Option,plotOption)
  [nD,nA]=size(MatDA);
Vp=find(MatDA==1);
Vn=find(MatDA==0);
Ip=crossvalind('Kfold',numel(Vp),K);
In=crossvalind('Kfold',numel(Vn),K);

MatPredict=zeros(nD,nA);
for I=1:K
        vp=Ip==I;
        vn=In==I;
        Mat=MatDA;
        Mat([Vp(vp);Vn(vn)])=0;
        Kd=projectNetworkSimilarity(Mat,toplogyOption);
        Ka=projectNetworkSimilarity(Mat',toplogyOption);
        if weightOption==1
              SD=weightCoef(1)*Sd+(1-weightCoef(2))*Kd;
              SA=weightCoef(1)*Sa+(1-weightCoef(2))*Ka;
        end
        if weightOption==2
                SD=Sd.^weightCoef(1).*Kd.^(1-weightCoef(2));
                SA=Sa.^weightCoef(1).*Ka.^(1-weightCoef(2));
        end
        matPredict=semi_supervisedModel3(Mat,SD,SA,alph,Option);

        MatPredict([Vp(vp);Vn(vn)])=matPredict([Vp(vp);Vn(vn)]);
end
[AUC,AUPR,Acc,Sen,Spe,Pre]=ROCcompute(MatPredict,MatDA,plotOption);
%%%
```

SemiSupervisedModelPredict.m

```
%This code is used to evaluate the performance of models built by SLP based on
%prospective evaluation;
function [AUC,AUPR,Acc,Sen,Spe,Pre,MatPredict]=SemiSupervisedModelPredict ...
(MatDA1,MatDA2,Sd,Sa,toplogyOption,weightOption,weightCoef,alph,Option, …
plotOption)
    Kd=projectNetworkSimilarity(MatDA1,toplogyOption);
    Ka=projectNetworkSimilarity(MatDA1',toplogyOption);
    if weightOption==1
        Sd=weightCoef(1)*Sd+(1-weightCoef(2))*Kd;
        Sa=weightCoef(1)*Sa+(1-weightCoef(2))*Ka;
    end
    if weightOption==2
        Sd=Sd.^weightCoef(1).*Kd.^(1-weightCoef(2));
        Sa=Sa.^weightCoef(1).*Ka.^(1-weightCoef(2));
    end
    MatPredict=semi_supervisedModel3(MatDA1,Sd,Sa,alph,Option);
    v=MatDA1==0;
    PredictV=MatPredict(v);
    TrueV=MatDA2(v);
    [AUC,AUPR,Acc,Sen,Spe,Pre]=ROCcompute(PredictV,TrueV,plotOption);
%%%
```

simBaseModelCVtest.m

```
%This code is used to evaluate the performance of models built by NN based on 10
% folds cross validation;
function [AUC,AUPR,Acc,Sen,Spe,Pre,MatPredict]=simBaseModelCVtest( ...
MatDA,K,Sd,Sa,toplogyOption,weightOption,weightCoef,plotOption)
[nD,nA]=size(MatDA);
Vp=find(MatDA==1);
Vn=find(MatDA==0);
Ip=crossvalind('Kfold',numel(Vp),K);
In=crossvalind('Kfold',numel(Vn),K);

MatPredictD=zeros(nD,nA);
MatPredictA=zeros(nD,nA);
for I=1:K
    vp=Ip==I;
    vn=In==I;
    Mat=MatDA;
    Mat([Vp(vp);Vn(vn)])=0;
    Kd=projectNetworkSimilarity(Mat,toplogyOption);
    Ka=projectNetworkSimilarity(Mat',toplogyOption);
```

```
        if weightOption==1
              SD=weightCoef(1)*Sd+(1-weightCoef(1))*Kd;
              SA=weightCoef(2)*Sa+(1-weightCoef(2))*Ka;
        end
        if weightOption==2
                SD=Sd.^weightCoef(1).*Kd.^(1-weightCoef(1));
                SA=Sa.^weightCoef(2).*Ka.^(1-weightCoef(2));
        end

        matPredict=simBaseModel(Mat,SD);
        MatPredictD([Vp(vp);Vn(vn)])=matPredict([Vp(vp);Vn(vn)]);
        matPredict=simBaseModel(Mat',SA);
        matPredict=matPredict';
        MatPredictA([Vp(vp);Vn(vn)])=matPredict([Vp(vp);Vn(vn)]);
end

MatPredict=max(MatPredictD,MatPredictA);
[AUC,AUPR,Acc,Sen,Spe,Pre]=ROCcompute(MatPredict,MatDA,plotOption);
%%%

simBaseModelPredict.m
%This code is used to evaluate the performance of models built by NN based on
% prospective evaluation;
function [AUC,AUPR,Acc,Sen,Spe,Pre,MatPredict]=simBaseModelPredict( …
MatDA1,MatDA2,Sd,Sa,toplogyOption,weightOption,weightCoef,plotOption)
        Kd=projectNetworkSimilarity(MatDA1,toplogyOption);
        Ka=projectNetworkSimilarity(MatDA1',toplogyOption);
        if weightOption==1
              Sd=weightCoef(1)*Sd+(1-weightCoef(1))*Kd;
              Sa=weightCoef(2)*Sa+(1-weightCoef(2))*Ka;
        end
        if weightOption==2
                Sd=Sd.^weightCoef(1).*Kd.^(1-weightCoef(1));
                Sa=Sa.^weightCoef(2).*Ka.^(1-weightCoef(2));
        end

        MatPredictD=simBaseModel(MatDA1,Sd);
        MatPredictA=simBaseModel(MatDA1',Sa);

      MatPredict=max(MatPredictD,MatPredictA');

      v=MatDA1==0;
      PredictV=MatPredict(v);
      TrueV=MatDA2(v);
```

```
[AUC,AUPR,Acc,Sen,Spe,Pre]=ROCcompute(PredictV,TrueV,plotOption);
```
